# Chronic obstructive pulmonary disease and exacerbations: Patient insights from the global Hidden Depths of COPD survey

**DOI:** 10.1186/1471-2466-13-54

**Published:** 2013-08-23

**Authors:** Neil Barnes, Peter MA Calverley, Alan Kaplan, Klaus F Rabe

**Affiliations:** 1 Department of Respiratory Medicine, London Chest Hospital (Barts Health NHS Trust), Bonner Road, London E2 9JX, UK; 2 Division of Infection and Immunity, Clinical Sciences Centre, University Hospital Aintree, Lower Lane, Liverpool L9 7AL, UK; 3 Canada and Bedford Park Family Medical Centre, University of Toronto, 17 Bedford Park Avenue, Richmond Hill, Ontario L4C 2N9, Canada; 4 Department of Medicine, Germany and LungenClinic Grosshansdorf, members of the German Center for Lung Research, University Kiel, D-22927, Grosshansdorf, Germany

**Keywords:** COPD, Exacerbation, Patient-reported, Survey

## Abstract

**Background:**

Although chronic obstructive pulmonary disease (COPD) is a major global health burden there is a lack of patient awareness of disease severity, particularly in relation to exacerbations.

**Methods:**

We conducted a global patient survey using an innovative, internet-based methodology to gain insight into patient perceptions of COPD and exacerbations in a real-world sample typical of today’s working-age COPD population.

**Results:**

Two thousand patients with COPD (53%), chronic bronchitis (52%) and/or emphysema (22%) from 14 countries completed an online questionnaire developed by the authors. The Medical Research Council (MRC) breathlessness scale was used to delineate symptom severity. Over three quarters of patients (77%) had experienced an exacerbation, with 27% of MRC 1 and 2 patients and 52% of MRC 3, 4 and 5 patients requiring hospitalization as a result of an exacerbation. While a majority of MRC 1 and 2 patients (51%) reported being back to normal within a few days of an exacerbation, 23% of MRC 3, 4 and 5 patients took several weeks to return to normal and 6% never fully recovered. A high proportion of patients (39%) took a ‘wait and see’ approach to exacerbations.

Despite the high prevalence of exacerbations and their negative impact on quality of life, 73% of MRC 1 and 2 patients and 64% of MRC 3, 4 and 5 patients felt that they had control of their COPD. However, 77% of all patients were worried about their long-term health, and 38% of MRC 1 and 2 patients and 59% of MRC 3, 4 and 5 patients feared premature death due to COPD.

**Conclusions:**

To reduce the adverse effects of COPD on patients’ quality of life and address their fears for the future, we need better patient education and improved prevention and treatment of exacerbations.

## Background

Chronic obstructive pulmonary disease (COPD) is a major global health burden in both developed and developing countries. The disease is predicted to become the third leading cause of worldwide disease burden by 2030 [1]. COPD is also the leading respiratory cause of days lost from work [2], and three quarters of COPD patients report difficulty in simple day-to-day activities such as dressing and walking up stairs [3].

Until recently, the major goal of COPD treatment was the reduction of symptoms. However, with the recognition that exacerbations of COPD are very common, have a major adverse impact on quality of life, and may speed disease progression, guidelines and clinical attention are focusing on reducing future risks, such as the prevention and treatment of exacerbations [4]. In developed countries the hospitalization of COPD patients, caused predominantly by exacerbations, accounts for more than 50% of direct healthcare costs [5].

Surveys of patients with COPD have found that there is a considerable burden of disease and that patients have a poor knowledge of COPD [6-9]. Furthermore, an international survey of 3,265 COPD patients revealed that many patients underestimate the severity of their disease [10]. However, these surveys have relied on relatively small sample sizes or have been predominantly focused on Europe and North America. Therefore, we aimed to gain a global insight into patients’ perceptions of COPD and, more uniquely, their exacerbations, in a real-world setting using an innovative, internet-based methodology. The survey was designed to identify differences and similarities in perceptions between patients of differing COPD severities, using the Medical Research Council (MRC) breathlessness scale to delineate severity [11].

## Methods

The survey was performed in 14 countries: Australia, Brazil, Canada, China, Denmark, France, Germany, Italy, the Netherlands, Poland, South Korea, Spain, Turkey and the UK. These countries were chosen to provide a wide geographic and economic spread.

An online approach was used to ensure that the methodology was globally consistent while avoiding the need to rely on treatment centres for recruitment and taking into account the difficulty in implementing a telephone survey in countries such as China and Brazil. The survey therefore avoided potential biases within specialist centres or regions as well as biases related to disease severity or treatment. This innovative, internet-based method, commonly used for consumer research, recruited participants from established online general population research panels containing over 18 million members worldwide. The research was implemented by professional market researchers (ICM Research) in accordance with the Legal and Ethical Guidelines issued by the British Healthcare Business Intelligence Association (BHBIA) and was conducted in accordance with codes of conduct regarding anonymity, confidentiality and ethical practice. The survey was therefore exempt from ethics approval under the UK Governance arrangements for research ethics committees.

**Figure 1:**
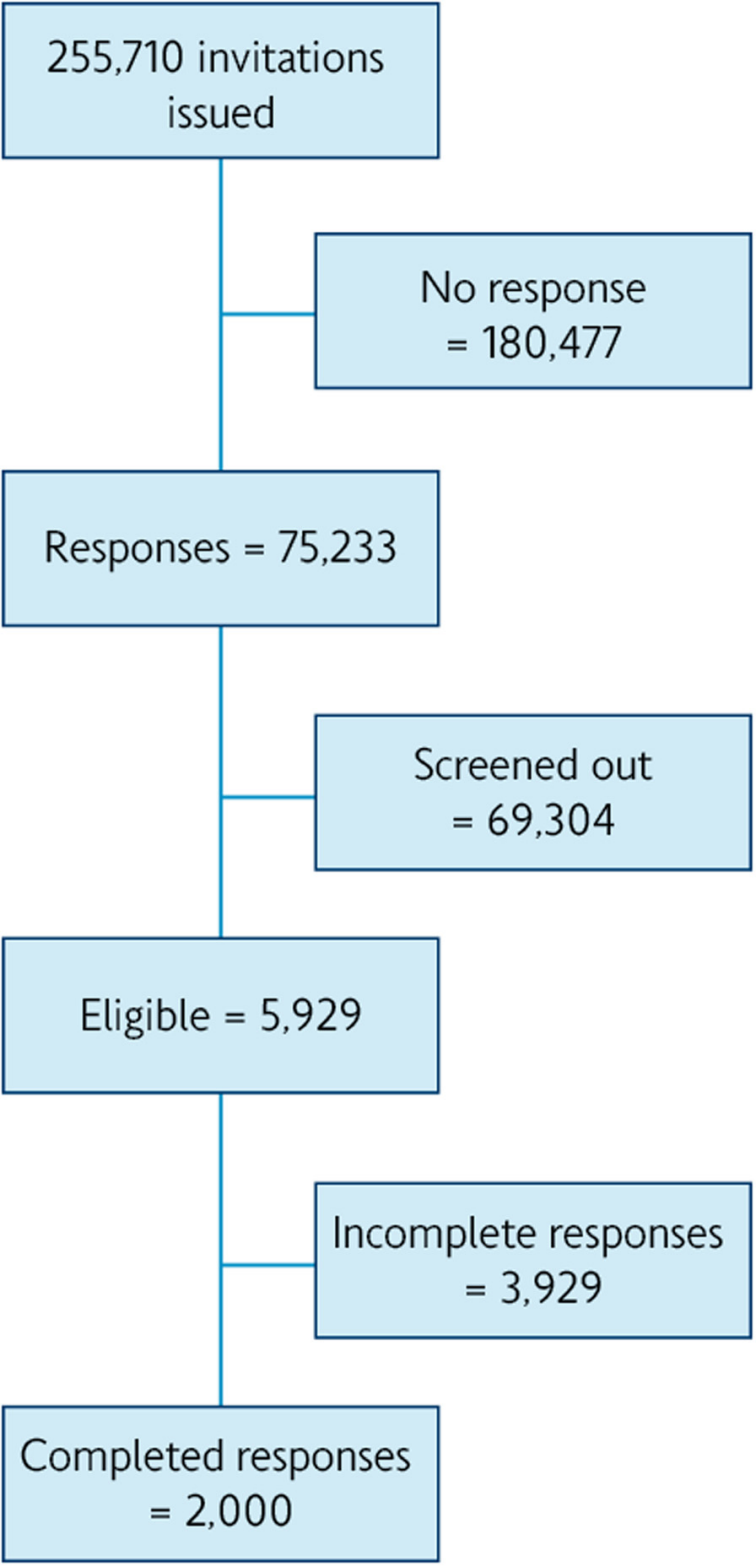
Flow diagram of patient selection.

Based on a self-reported respiratory condition/breathing problem and/or a positive current or former smoking history, 255,710 individuals were invited to participate in the survey between 09 July and 02 September 2010. Information about the survey, which included the length of time for completion of the questionnaire (approximately 17 minutes), was e-mailed to the invitees. Incentives were offered in line with the terms and conditions of the panels, and were often non-monetary, or ranged from the equivalent of £0.20 to £1 per minute of survey. Of 255,710 invitees, 75,233 responded and, after providing consent, were screened for eligibility, producing 5,929 respondents who were able to withdraw at any point. A sample size of 2,000 completed questionnaires was used for analysis (Figure 1).

All patients who took part needed to have been diagnosed by a clinician with one or more of the following conditions: COPD, chronic bronchitis or emphysema. Patients also needed to have at least two of the following symptoms: breathlessness on exertion, mucus/sputum/phlegm production, chronic or troublesome cough, chest pain when walking, regular chest infections (especially in the winter) or leg pain when walking. Disease severity was measured by asking patients to assess themselves according to the criteria of the MRC breathlessness scale (Grades 1–5) [11]. A symptom-based definition of exacerbations was used (a worsening of at least one symptom of COPD lasting for at least 48 hours) [12], which was outlined for patients each time there was a relevant question.

The survey consisted of a two-part, online, self-completion questionnaire that used an adaptive question approach to minimize unnecessary questions and shorten completion times. The questions were developed by the authors using standard measures where appropriate (for example, the MRC breathlessness scale). The questionnaire and the screening approach can be viewed in the online supplement.

The survey was tested by three individuals prior to launch, and was then ‘soft-launched’ to a limited number of respondents (50–100 per country) so that the data and survey mechanism could be tested for sense/logic, and the average time for completion checked against the original estimate. As some questions were open-ended, completed questionnaires could contain unanswered questions, and checks were only made to ascertain whether text was entered. In addition, respondents were offered a “don’t know” answer where applicable to avoid forcing inaccurate responses. Data from incomplete questionnaires were collected but not processed or analysed.

No analytical time stamps were used but all data were logic tested to ensure that respondents did not provide contradictory answers. Quality control questions were included at the beginning and end of the questionnaire. These asked personal information, for example, age at the beginning of the questionnaire and date of birth at the end, and if these answers did not match, the respondent’s questionnaire was rejected.

Data were stored in compliance with the UK Data Protection Act (1998) on secure servers that could be accessed only by relevant researchers, and each respondent was issued with a unique Uniform Resource Locator (URL) that could be used once to access the questionnaire. Respondents were not able to review or edit their answers to previous questions.

The current publication was developed in line with the Checklist for Reporting Results of Internet E-Surveys (CHERRIES) [13].

### Statistical analysis

The collected data were processed and tabulated into electronic data tables. Descriptive statistics are presented herein.

### Role of the funding source

The study was sponsored by Nycomed (a Takeda company). A Steering Committee of COPD experts including primary and secondary care physicians designed the survey in conjunction with six representatives of the sponsor. This included the original study design and concept, the plan for the analyses, full access to the data and responsibility for decisions with regard to publication. The research was implemented by professional market researchers (ICM Research).

## Results

The 2,000 completed questionnaires consisted of 150 questionnaires from each country except for Denmark and Turkey, where limited numbers of individuals in consumer research panels meant that only 100 completed questionnaires in each of these countries were gathered. The mean age (standard error [SE]) of the population was 52.99 (0.22) years, 53% of patients were current smokers, 1,231 (62%) patients were classified as MRC breathlessness scale 1 and 2, and 769 (38%) patients were classified as MRC breathlessness scale 3, 4 and 5 (Table 1). The UK had the highest percentage (58%) and Italy the lowest percentage (22%) of MRC 3, 4 and 5 patients (Table 1). Symptoms such as breathlessness on exertion, fatigue, sputum production and cough were very common (Table 1). Regular chest infections were experienced by 41% of MRC 1 and 2 patients and 57% of MRC 3, 4 and 5 patients. The majority of patients (69%) thought that their COPD was controlled, yet MRC 1 and 2 patients and MRC 3, 4 and 5 patients reported a mean (SE) of 10 (0.27) and 18 (0.37) days per month, respectively, in which COPD negatively affected their life. The majority of patients felt that their doctor took their disease seriously or very seriously, with only a minority (5% of all patients) feeling that their doctor did not take their condition seriously at all (Table 1).

**Table 1:** **Patient demographics**, **symptoms and perceptions**

	**MRC 1 and 2**	**MRC 3, 4 and 5**
**N (%)**	1,231 (62%)	769 (38%)
**Male, n (%)**	656 (53%)	392 (51%)
**Female, n (%)**	575 (47%)	377 (49%)
**Mean age, years (SE)**	52.03 (0.28)	54.54 (0.35)
MRC breathlessness scale by country, n (%)*		
Australia	84 (56%)	66 (44%)
Brazil	106 (71%)	44 (29%)
Canada	70 (47%)	80 (53%)
China	110 (73%)	40 (27%)
Denmark	50 (50%)	50 (50%)
France	79 (53%)	71 (47%)
Germany	84 (56%)	66 (44%)
Italy	113 (75%)	37 (25%)
The Netherlands	94 (63%)	56 (37%)
Poland	98 (65%)	52 (35%)
South Korea	101 (67%)	49 (33%)
Spain	107 (71%)	43 (29%)
Turkey	72 (72%)	28 (28%)
The UK	63 (42%)	87 (58%)
**Have you ever taken a pulmonary function test? n (%)**		
Yes	960 (78%)	678 (88%)
No	223 (18%)	64 (8%)
**Current smoking behaviour**		
More than 20 cigarettes per day, n (%)	136 (11%)	91 (12%)
Up to 20 cigarettes per day, n (%)	536 (44%)	295 (38%)
Former smoker, n (%)	396 (32%)	307 (40%)
Never smoked, n (%)	163 (13%)	76 (10%)
**Days negatively affected by COPD in a 30 day month, mean days (SE)**	10 (0.27)	18 (0.37)
**COPD symptoms experienced, n (%)**		
Breathlessness on exertion	958 (78%)	704 (92%)
Fatigue	752 (61%)	606 (79%)
Mucus/sputum/phlegm production	731 (59%)	505 (66%)
Chronic/troublesome cough	729 (59%)	484 (63%)
Regular chest infections especially in winter	500 (41%)	439 (57%)
Leg pain on walking	331 (27%)	340 (44%)
Chest pain on walking	218 (18%)	277 (36%)
Other	24 (2%)	15 (2%)
**How seriously does your doctor take your COPD? n (%)**		
Very seriously	297 (24%)	331 (43%)
Fairly seriously	480 (39%)	303 (39%)
Not particularly seriously	347 (28%)	107 (14%)
Not at all seriously	51 (4%)	11 (1%)
**How well do patients think their COPD is controlled? n (%)**		
Not at all well	47 (4%)	54 (7%)
Not particularly well	255 (21%)	204 (27%)
Quite well	718 (58%)	443 (58%)
Very well	178 (14%)	51 (7%)

MRC=Medical Research Council breathlessness scale; SE=standard error; COPD=chronic obstructive pulmonary disease.

*N=150 for each country except for Denmark and Turkey where N=100.

**Figure 2:**
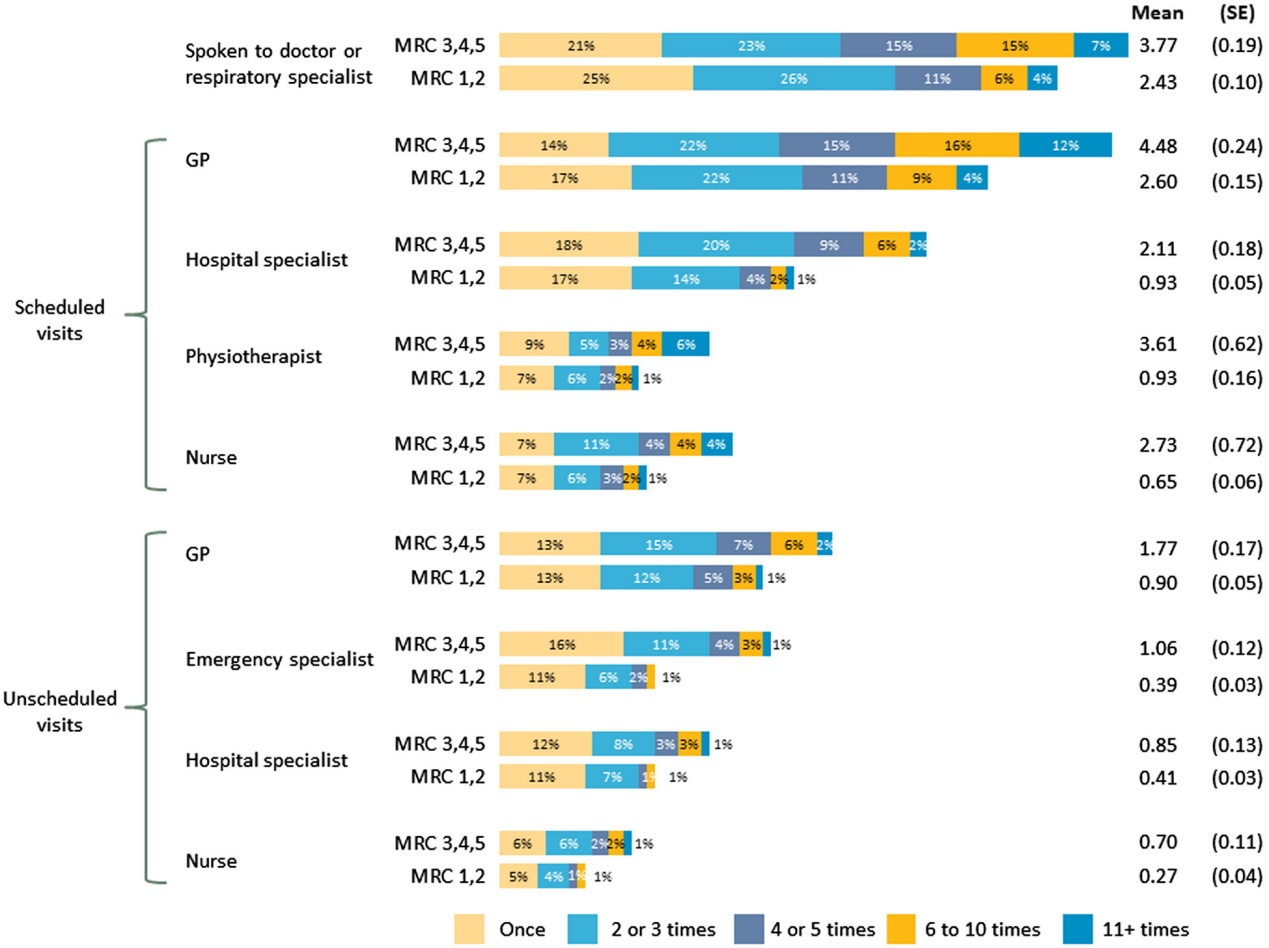
Healthcare utilization in the preceding 12 months.

Patients reported high healthcare utilization in the preceding 12 months of the survey (Figure 2). This included high frequencies of scheduled and unscheduled visits to primary care physicians, specialists and allied healthcare professionals such as physiotherapists (Figure 2). Use of unscheduled healthcare was particularly common among MRC 3, 4 and 5 patients, with a per-year mean (SE) of 1.77 (0.17) unscheduled GP visits, 0.85 (0.13) unscheduled visits to hospital specialists and 0.70 (0.11) unscheduled visits to a nurse. MRC 3, 4 and 5 patients also reported a per-year mean (SE) of 1.06 (0.12) unscheduled visits to the emergency department, with 19% of these patients reporting two or more visits.

**Figure 3:**
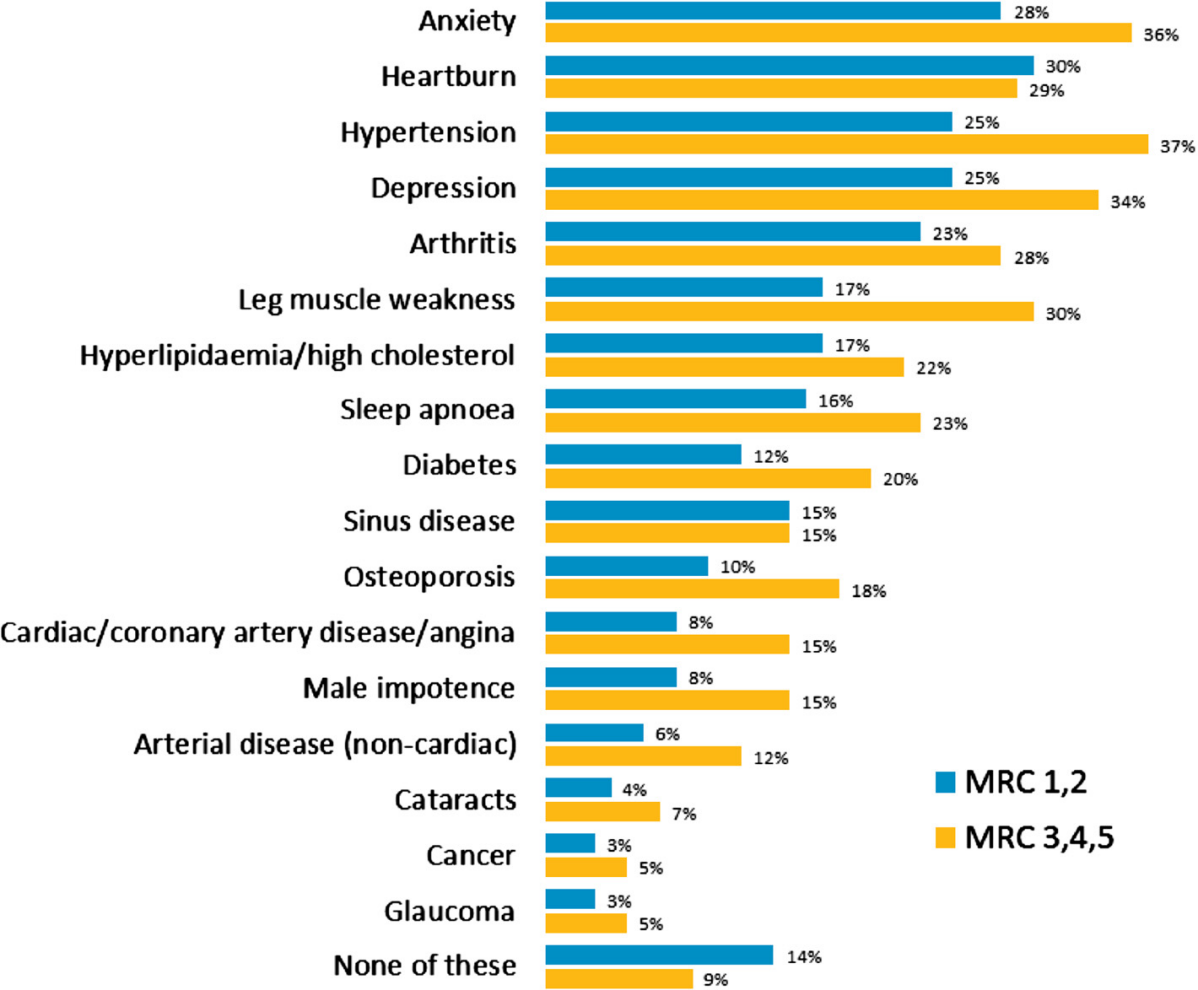
Comorbidities.

Self-reported comorbidities were generally more common in MRC 3, 4 and 5 patients compared with MRC 1 and 2 patients, and included hypertension (37%), anxiety (36%), depression (34%), leg muscle weakness (30%), heartburn (29%), arthritis (28%), hyperlipidaemia/high cholesterol (22%), sleep apnoea (23%) and diabetes (20%) (Figure 3).

Prescription medication was used by a high percentage of all patients (89%), with bronchodilators the mainstay of treatment, as per COPD guidelines (Table 2) [4]. Over a quarter (27%) of patients had taken steroids, and 43% of patients had used antibiotics. Nearly a quarter (23%) of MRC 3, 4 and 5 patients had taken oxygen. Patients commonly increased medication use with worsening symptoms (Table 2). Lifestyle choices for managing COPD, such as quitting smoking, exercising and eating more healthily, were also common (Table 2).

**Table 2:** Treatments and lifestyle choices for managing COPD

	**MRC 1 and 2**	**MRC 3, 4 and 5**
	**(n=1,231)**	**(n=769)**
**Treatments and lifestyle choices for managing COPD, n (%)**		
Long-acting bronchodilators	710 (58%)	582 (76%)
Short-acting bronchodilators	390 (32%)	406 (53%)
Antibiotics	494 (40%)	358 (47%)
Steroids (all types)	267 (22%)	273 (36%)
Other prescription medication	179 (15%)	168 (22%)
Natural remedies and/or alternative medicine	232 (19%)	147 (19%)
Quit smoking	509 (41%)	365 (47%)
Cutting down on smoking	480 (39%)	286 (37%)
Breathing exercise	405 (33%)	367 (48%)
Eating healthier/better diet	445 (36%)	320 (42%)
Physical exercise	455 (37%)	251 (33%)
Oxygen	119 (10%)	176 (23%)
Pulmonary rehabilitation	87 (7%)	136 (18%)
**Treatments and lifestyle choices used more by patients during COPD symptom worsening, n (%)**		
Long-acting bronchodilators	854 (69%)	522 (68%)
Short-acting bronchodilators	339 (28%)	294 (38%)
Antibiotics	228 (19%)	179 (23%)
Steroids (all types)	202 (16%)	168 (22%)
Other prescription medication	118 (10%)	85 (11%)
Natural remedies and/or alternative medicine	187 (15%)	95 (12%)
Quit smoking	182 (15%)	104 (14%)
Cutting down on smoking	278 (23%)	150 (20%)
Breathing exercise	273 (22%)	205 (27%)
Eating healthier/better diet	203 (16%)	126 (16%)
Physical exercise	217 (18%)	113 (15%)
Oxygen	170 (14%)	133 (17%)
Pulmonary rehabilitation	118 (10%)	82 (11%)

MRC=Medical Research Council breathlessness scale; COPD=chronic obstructive pulmonary disease.

**Figure 4:**
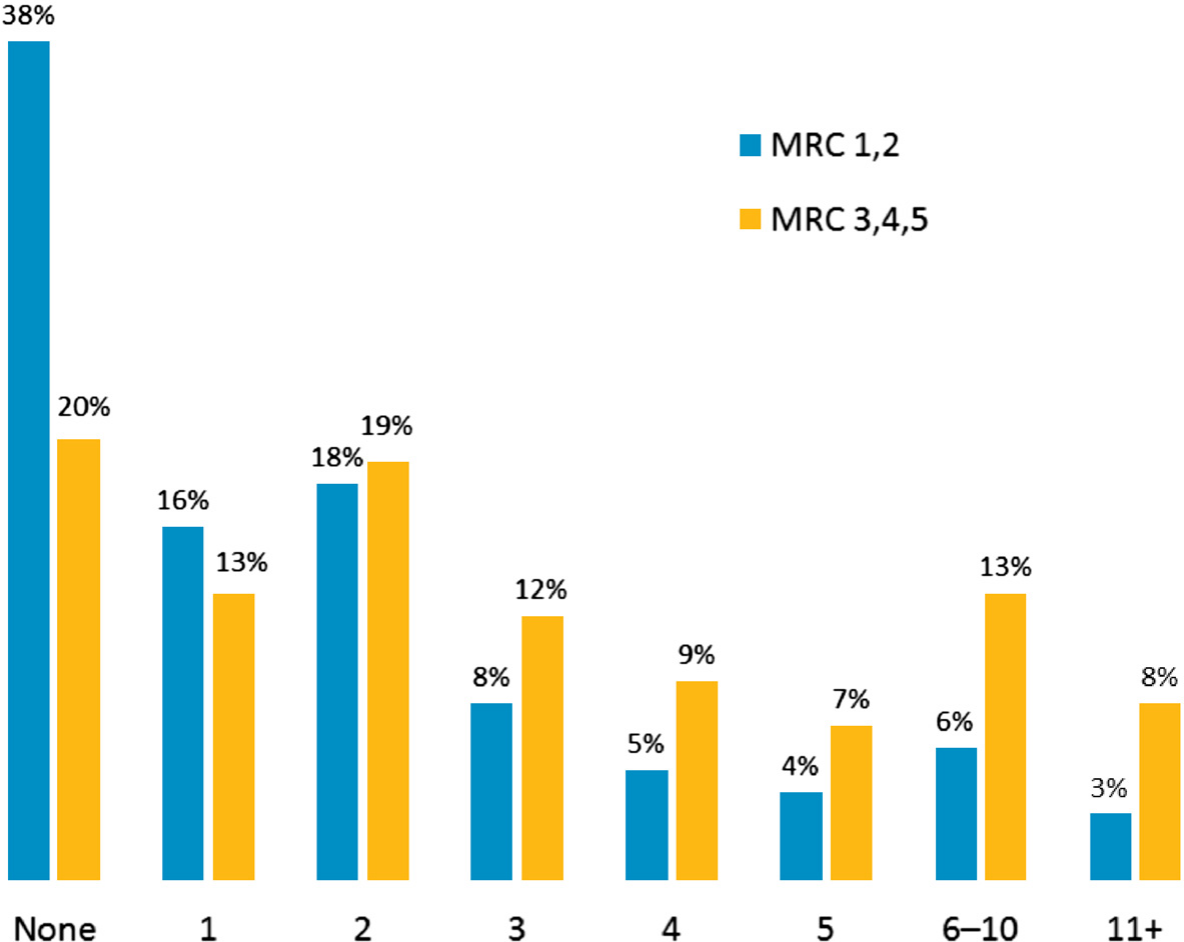
Frequency of exacerbations in the preceding 12 months.

Over three quarters (77%) of all patients had experienced an exacerbation (Table 3). The proportion of patients reporting exacerbations in the preceding year was also high: 62% and 80% for MRC 1 and 2 patients and MRC 3, 4 and 5 patients, respectively. A high percentage of patients had had two or more exacerbations in the preceding 12 months (Figure 4). Over a half (53%) of MRC 3, 4 and 5 patients had experienced an exacerbation that required hospitalization (Table 3). While a majority of MRC 1 and 2 patients (51%) reported being back to normal within a few days of an exacerbation, 23% of MRC 3, 4 and 5 patients took several weeks to return to normal and 6% never fully recovered. A high proportion of patients (39%) took a ‘wait and see’ approach to exacerbations (Table 3).

**Table 3:** Exacerbations and psychosocial impact

	**MRC 1 and 2**	**MRC 3, 4 and 5**
	**(n=1,231)**	**(n=769)**
**Proportion of patients with an exacerbation, n (%)**		
Ever	875 (71%)	659 (86%)
In the last 12 months	760 (62%)	617 (80%)
**Proportion of people hospitalised by an exacerbation (baseline: n=875 & 659), n (%)**	239 (27%)	342 (52%)
**Time taken to return to normal after an exacerbation (baseline: n=875 & 659), n (%)**		
Within a few days	450 (51%)	266 (40%)
Within a week	255 (29%)	169 (26%)
Within a few weeks	104 (12%)	150 (23%)
Within a month	28 (3%)	18 (3%)
Within a few months	16 (2%)	14 (2%)
Longer	7 (1%)	3 (0%)
Never	15 (2%)	39 (6%)
**Patient reaction to the onset of an exacerbation (baseline: n=875 & 659), n (%)**		
Take action right away	493 (56%)	379 (58%)
Wait and see	345 (39%)	246 (37%)
Do nothing	37 (4%)	34 (5%)
**Patient concern for long-term health as a consequence of having COPD, n (%)**		
Not at all worried	38 (3%)	16 (2%)
Not particularly worried	112 (9%)	25 (3%)
Neither worried nor unworried	167 (14%)	94 (12%)
Somewhat worried	671 (55%)	358 (47%)
Extremely worried	236 (19%)	271 (35%)
**Patient fear of premature death from COPD, n (%)**		
Not at all scared	171 (14%)	59 (8%)
Not particularly scared	536 (44%)	230 (30%)
Quite scared	368 (30%)	319 (41%)
Very scared	101 (8%)	134 (17%)
**Fear of premature death from an exacerbation (baseline: n=875 & 659), n (%)**		
Not at all scared	110 (13%)	47 (7%)
Not particularly scared	327 (37%)	176 (27%)
Quite scared	305 (35%)	267 (41%)
Very scared	104 (12%)	149 (23%)

MRC=Medical Research Council breathlessness scale; COPD=chronic obstructive pulmonary disease.

**Figure 5:**
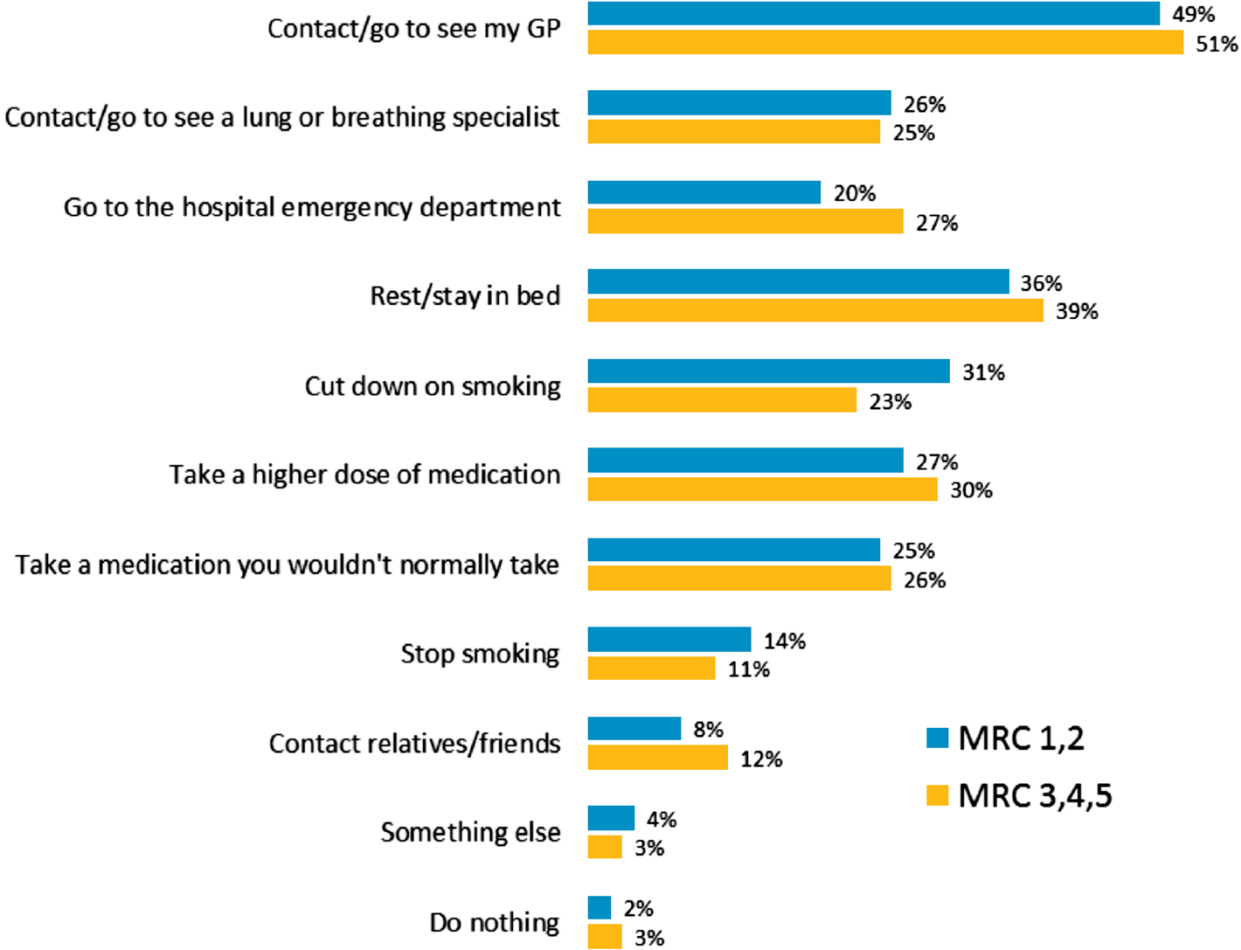
Response to an exacerbation.

**Figure 6:**
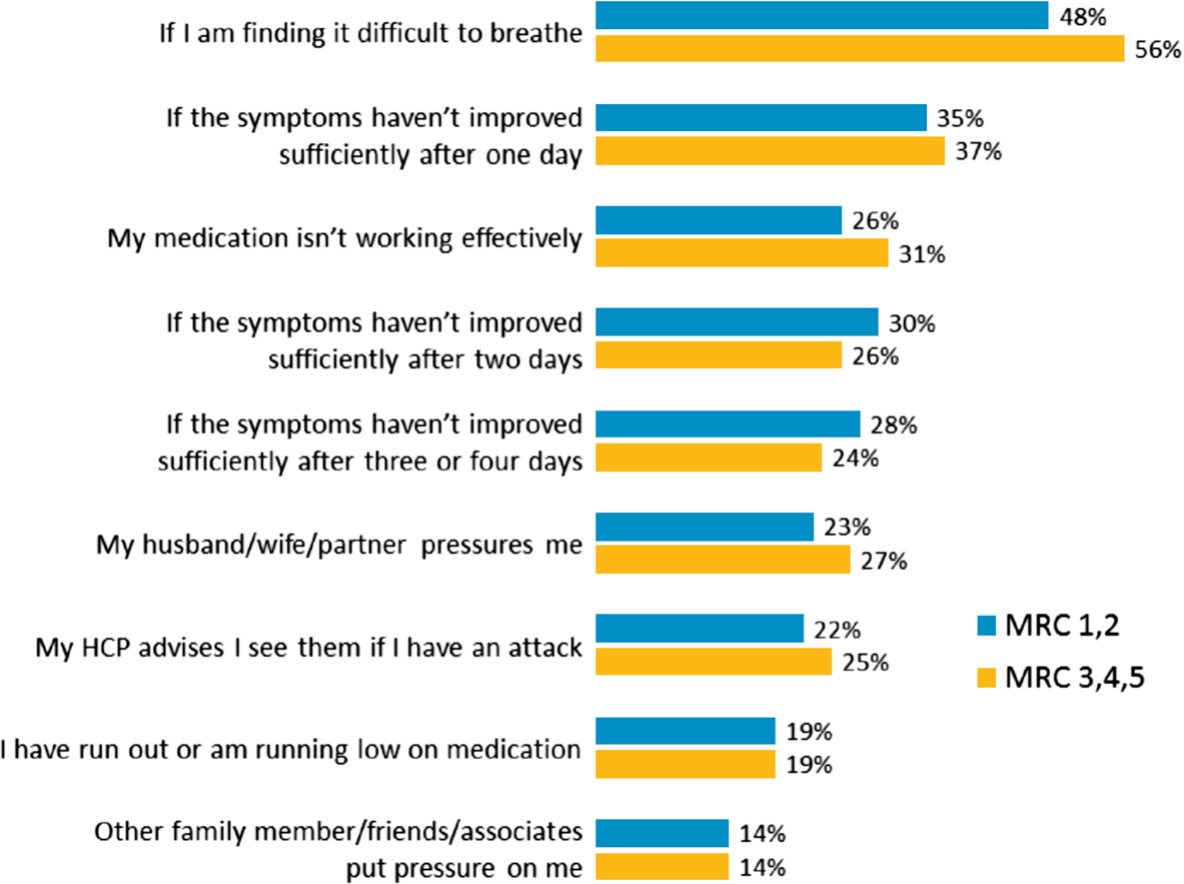
**Treatment**-**seeking triggers during an exacerbation.**

Nearly three quarters (73%) of patients contacted their healthcare service during an exacerbation (Figure 5). Other patient reactions to an exacerbation included rest, cutting down on smoking, taking higher doses of medication and taking a medication that would not be part of their usual regimen (Figure 5). Common reasons for seeking healthcare during an exacerbation were an increase in breathlessness, symptoms not improving sufficiently and ineffective medication (Figure 6).

**Figure 7:**
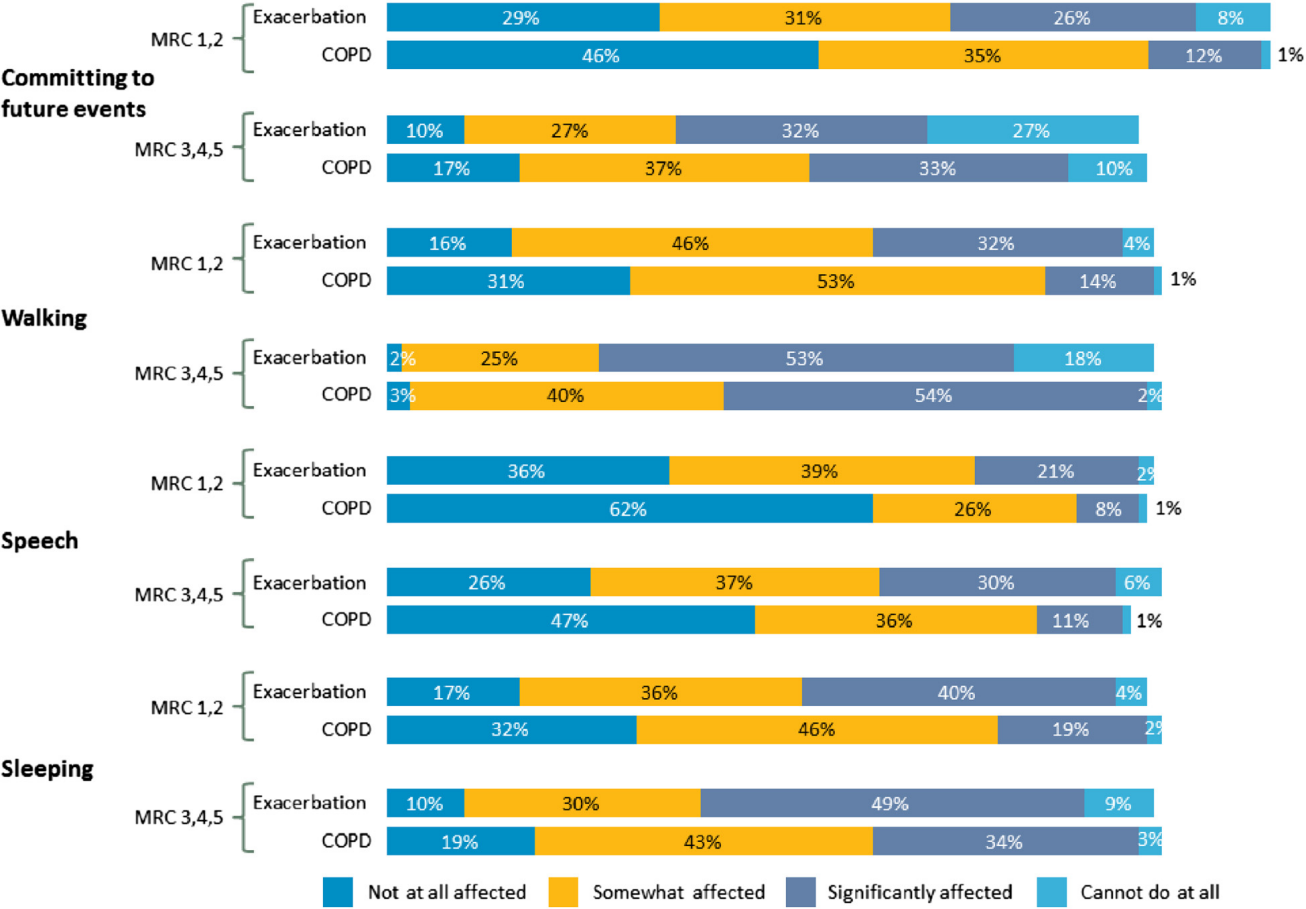
Impact of COPD and exacerbations.

Patients felt that COPD and exacerbations affected their quality of life and the ability to commit to future events (Figure 7). Over three quarters (77%) of all patients were worried about their long-term health, and 38% of MRC 1 and 2 patients and 59% of MRC 3, 4 and 5 patients feared premature death due to COPD (Table 3).

## Discussion

Our survey provides a unique, global perspective of how COPD patients perceive their illness and its impact on their everyday lives, with a focus on patient attitudes and responses to exacerbations. Recruitment via online research panels aimed to identify ‘real-world’ COPD patients whose views and experiences of the condition were typical of a wider COPD population. Online recruitment had the advantage of assessing a wide cross-section of the population but had the disadvantage of only assessing individuals with access to the internet who were motivated to respond. Therefore, the data herein may under-represent older individuals and over-represent more symptomatic individuals who were motivated to respond. However, the age, gender balance, smoking prevalence, symptom reporting, comorbidities and treatment history of the group suggest that respondents were indeed a representative COPD population.

With a mean age of 53 years, the survey cohort was already experiencing regular exacerbations, a considerable impact on daily functioning and high levels of healthcare utilization. These findings support those of other studies that show that the impact of COPD is not restricted to an elderly population [8,14,15]. Indeed, an analysis of data from the European Community Respiratory Health Survey of over 18,000 adults aged 20–44 years concluded that a considerable percentage of the population showed signs of COPD (11.8% GOLD stage 0, 2.5% GOLD stage 1 and 1.1% GOLD stages 2 and 3) [15].

The incidence of comorbidities reported here is likely associated with the age and smoking characteristics of the population – over half of patients in our survey were current smokers. Comorbidities were common, and were generally similar to those reported by another survey of patients with COPD [9], the Evaluation of COPD Longitudinally to Identify Predictive Surrogate Endpoints (ECLIPSE) data [16], and the comorbidities of COPD patients in The Health Improvement Network (THIN) database [17]. However, the rates of comorbidities in our survey were generally lower than those reported by other studies [9,16,17]. Furthermore, rates of comorbidities were generally higher in MRC 3, 4, and 5 patients compared with MRC 1 and 2 patients in our survey, contrasting with the ECLIPSE study that reported no relationship between comorbidity prevalence and COPD severity. The younger mean age of our survey population compared with the other studies, and inclusion of patients with chronic bronchitis and/or emphysema in our survey, may explain the differing comorbidity observations.

Treatment history was typical of a COPD population, and showed a relatively positive infiltration of guideline messages and an encouraging level of physical management approaches. The COPD Resource Network Needs Assessment Survey reported both patient and physician confusion about COPD treatment choices, and under-use of pulmonary rehabilitation [6]. In our survey, patients reported using physical and breathing exercises, but low rates of pulmonary rehabilitation, suggesting that better access to this treatment approach is still needed.

Patients generally believed that their COPD was well controlled despite the high rate of exacerbations and resulting need to consult healthcare services, rest and increase their medication. This type of mismatch is not unusual, and has been widely reported in studies of both COPD and asthma patients, suggesting low levels of expectation [6,10,18]. For example, the Confronting COPD International Survey, the first large international (EU and US) survey on the burden of COPD, reported that over a third of patients with the most severe breathlessness (too breathless to leave the house) described their condition as mild or moderate, as did 60% of patients characterised as breathless after walking a few minutes on level ground [10]. Similarly, in the COPD Resource Network Needs Assessment Survey, the majority of patients expressed satisfaction with their care despite experiencing significant symptoms and high healthcare utilization [6].

The MRC breathlessness scale proved a useful self-assessment indicator of COPD severity in our survey, with a consistent association between higher MRC scale (3, 4 and 5) and increased prevalence of exacerbations and symptoms, increased prescribed medication use and greater healthcare utilization. In addition, nearly twice as many MRC 3, 4 and 5 patients reported that their doctor took their condition very seriously compared with MRC 1 and 2 patients.

Patient reporting of COPD exacerbations is a relatively reliable measure of true exacerbation frequency, with a good correlation between patient recall of the number of exacerbations and documented occurrence of exacerbations [19,20]. Furthermore, the high prevalence of exacerbations reported in our survey is consistent with those in other studies of COPD patient reports [7,20-22]. For example, in the Perception of Exacerbations of Chronic Obstructive Pulmonary Disease (PERCEIVE) survey, 89% of patients reported at least one episode of ‘flare-up’ of symptoms during the preceding year [7]. Patient-reported exacerbation rates are typically higher than those reported in clinical trials (as very unstable patients are not recruited into clinical trials, and because the patient definition of an exacerbation may not be the same as that used in clinical trials), and suggest that the ‘real-world’ experience of COPD patients is different from that of patients in a research setting. The inclusion of approximately twice as many patients with chronic bronchitis compared with patients with emphysema in our survey may contribute to the relatively high prevalence of exacerbations and relative paucity of comorbidities in our survey.

Our survey cohort reported that recovery from exacerbations could be slow or incomplete, especially for MRC 3, 4 and 5 patients. Again, this supports data from clinical studies that demonstrated incomplete recovery 35 days after exacerbation in approximately a quarter of patients [12,23]. The high levels of healthcare utilization reported in our survey are also similar to those reported by other studies. In PERCEIVE, 89% of patients who had experienced an exacerbation needed to see their doctor, and 21% required hospital admission [7]. Exacerbations generated a mean (standard deviation) of 5.1 (4.6) visits to the doctor per patient per year [7].

In a cohort of 128 patients with COPD, earlier treatment of exacerbations was associated with faster recovery (regression coefficient 0.42 days/day delay of treatment; confidence interval, 0.19–0.65; p<0.001), and failure to report exacerbations was associated with an increased risk of emergency hospitalization (Spearman's rank correlation coefficient=0.21, p=0.04) [24]. As over a third of patients in our survey took a ‘wait and see’ approach to exacerbations, there is a clear need for better patient education that stresses the importance of a rapid response to symptoms of an exacerbation.

In our survey, exacerbations impacted everyday activities such as sleeping, walking and the ability to commit to future events. For COPD patients of working age, such as those in our cohort, there are additional concerns. For example, a survey of 2,426 COPD patients aged 45–68 years revealed that nearly one in five patients was forced to retire prematurely because of their condition [8]. In addition, patients expressed concern about their ability to maintain their lifestyle and plan for the future [8]. Patients in our survey expressed similar concerns about their future health, as well as fears of premature death arising from COPD, especially as a result of an exacerbation. Palliative care is an important component in the treatment of COPD patients, particularly those with severe disease [25], but access remains poor [26]. Current guidelines recommend that clinicians initiate discussions about end-of-life care with appropriate patients [4,27].

## Conclusions

Our global survey – carried out almost a decade after the Global Initiative for Chronic Obstructive Lung Disease (GOLD) published its first consensus report on the diagnosis, management and prevention of COPD [28] – has shown that exacerbations remain a major burden to COPD patients and their families, and put a considerable demand on healthcare services. Furthermore, exacerbations may be more common in a ‘real-world’ COPD population compared with those in clinical trials.

Our survey has also shown that there is a mismatch between patient perceptions of COPD and the reality of their frequent exacerbations, impaired quality of life and fears for the future. In addition, a high proportion of patients were unaware of the importance of a rapid response to exacerbations, which may be necessary to achieve early and complete resolution of symptoms and recovery of lung function.

By highlighting the fears and concerns of COPD patients, many of whom are of working age with financial and familial responsibilities, the survey draws attention to the need for better patient education regarding the severity of the disease, the importance of prompt treatment of exacerbations, and the treatment and lifestyle options available.

## Abbreviations

CHERRIES: Checklist for Reporting Results of Internet E-Surveys; COPD: Chronic obstructive pulmonary disease; ECLIPSE: Evaluation of COPD Longitudinally to Identify Predictive Surrogate Endpoints; EU: European Union; GOLD: Global Initiative for Chronic Obstructive Lung Disease; MRC: Medical Research Council; PERCEIVE: Perception of Exacerbations of Chronic Obstructive Pulmonary Disease Survey; SD: Standard deviation; SE: Standard error; THIN: The Health Improvement Network; UK: United Kingdom; URL: Uniform resource locator; US: United States.

## Competing interests

N Barnes has received honoraria for giving talks for the following companies: GlaxoSmithKline, AstraZeneca, Chiesi Pharmaceuticals, Boehringer Ingelheim, Teva and Takeda/Nycomed.

PMA Calverley has served on Scientific Advisory Boards of AstraZeneca, Boehringer Ingelheim, GlaxoSmithKline, Novartis and Takeda/Nycomed, and has received research funding from AstraZeneca, Boehringer Ingelheim, GlaxoSmithKline and Takeda/Nycomed.

A Kaplan has served on advisory boards for Boehringer Ingelheim, AstraZeneca, Takeda/Nycomed, Graceway, Novartis, Pfizer and Purdue. He has received honoraria for giving talks for the above companies and GlaxoSmithKline, Merck Frosst, Sanofi and Ortho Janssen.

KF Rabe has received research funding from Altana Pharma, Novartis, AstraZeneca, MSD and Takeda/Nycomed. He has also provided consultation services for AstraZeneca, Chiesi Pharmaceuticals, Novartis, MSD and GlaxoSmithKline.

The study was sponsored by Nycomed (a Takeda company). The research was implemented by professional market researchers (ICM Research).

## Authors’ contributions

All authors have made substantial intellectual contributions to the conception and design of the study and the analysis and interpretation of the data. All authors have been involved in drafting the manuscript or revising it critically for important intellectual content.

## Pre-publication history

The pre-publication history for this paper can be accessed here:

http://www.biomedcentral.com/1471-2466/13/54/prepub
